# Emotionally Based School Avoidance (EBSA): Students’ Views of What Works in a Specialist Setting

**DOI:** 10.5334/cie.38

**Published:** 2022-05-18

**Authors:** Cathleen Halligan, Sarah Cryer

**Affiliations:** 1 London Borough of Camden, GB

**Keywords:** Emotionally based school avoidance, school attendance, specialist setting

## Abstract

“Emotionally based school avoidance” (EBSA) is a term used to describe young people who have difficulty attending school due to emotional needs. In comparison to previously favoured terms such as “school refuser”, EBSA highlights the impact of unmet emotional needs over school non-attendance, which then informs the intervention offered for students struggling to attend school. This paper presents an exploratory single-case study undertaken at a specialist GCSE setting (School X) for students experiencing EBSA. The work was commissioned by the programme following three consecutive years in which all students completing their GCSEs (national curriculum) showed improvement in attendance and 85% achieved above their predicted grade. In addition, 95% of students were still in post-16 study after leaving the school. The study, therefore, aimed to explore students’ views of protective factors in a setting where they have previously made progress in terms of attendance and achievement.

Qualitative data were gathered using semi-structured questions with students in a group setting, delivered online using an anonymised computer software system. Quantitative data were gathered with students in a one-to-one situation using an adaption of the Q-sort technique, a self-contained “qualiquantilogical” methodology that aims to explore human subjectivity. Findings were collectively analysed using thematic analysis, which produced two over-arching themes: interconnectivity and psychological safety. Findings from this study are considered alongside research about interventions suggested to be effective for supporting students experiencing EBSA to re-engage with school and education.

“Emotionally based school avoidance” (EBSA) is a term used to describe children and young people who frequently do not attend school due to emotional and mental health needs (West Sussex EPS, 2018). Research has made a distinction between students who are absent from school due to truancy and those who struggle with school attendance due to emotional distress (Thambirajah et al., 2008); therefore, the terminology of “school avoidance” is currently favoured over the previously commonly used “school refusal.” Some local authorities also refer to this as “persistent school non-attendance” to avoid locating the attendance issues within the young person (Tobias, 2019). For the purposes of this study, the decision was made to use the term “emotionally based school avoidance” to highlight the emotional difficulties that underpin attendance problems. This change in language signals a shift from viewing the avoidance of school as a choice made by the young person to focus more on understanding the underlying causes of school avoidance, which can then inform the interventions that schools and families put into place (London Borough of Camden, 2021; West Sussex EPS, 2018).

It has been suggested that EBSA affects approximately 1–2% of the UK school population (Elliott, 1999), but the prevalence rate is slightly higher when students reach secondary school (Gulliford & Miller, 2015). To date, there is little evidence of different prevalence rates among males and females and students from different socioeconomic backgrounds (King & Bernstein, 2001; Pelligrini, 2007).

Students experiencing EBSA experience a range of difficulties beyond school. Specifically, the literature suggests that EBSA negatively impacts academic attainment and leads to fewer social and, eventually, employment opportunities in adult life (Garry, 1996; Pellegrini, 2007; Taylor, 2012). Links have also been made between EBSA and significant adult mental health difficulties, including depression and anxiety (Planty et al., 2005; Walter et al., 2010). In addition, suicidal thoughts have been associated with difficulties with school attendance (Bjarnason & Thorlindsson, 1994).

## Contributing Factors

Thambirajah et al. (2008) emphasized that it is important to understand the underlying emotional factors that contribute to school non-attendance rather than solely considering student behaviours. For example, it has been suggested that students who are frequently absent from school experience feelings of anxiety (Finning et al., 2019). It is also important to consider the young person’s perceived ability to cope socially and academically as negative thoughts create additional anxiety and, therefore, further difficulties with avoidance of school (Heyne & Rollings, 2002). When in an anxious state, it is common for young people to use the fight, flight, or freeze survival response to protect themselves from the perceived threat; in this case, school (Frydman & Mayor, 2017). Avoidance of school can, therefore, be seen as a neurological stress response to the perceived threat of the school environment. These avoidance behaviours may allow students to feel a sense of control over a situation that they otherwise feel they have little agency over (Thambirajah et al., 2008), making it important to view EBSA in terms of the emotional needs that contribute to it.

According to Thambirajah et al. (2008), “school refusal occurs when … ‘pull’ factors that promote school non-attendance overcome the ‘push’ factors that encourage attendance” (p. 33). As a result, these authors advocate for exploring the systems around the child when planning interventions to improve attendance. Similarly, Southwell (2006) cautioned against placing issues of non-attendance within the children themselves and, instead, noted that school systems are key to understanding attendance issues.

Local authority educational psychology services (such as West Sussex and London Camden) have incorporated the concept of “push” and “pull” factors away from school and towards home into intervention planning for students experiencing EBSA (see London Borough of Camden EPS, 2021; West Sussex EPS, 2018). For example, unstable peer relationships may be a factor pushing young people away from school whereas parental illness may be a factor pulling the young person towards staying at home. Such insights can be used to develop support plans for students. Moreover, research has demonstrated that it is possible to reduce attendance difficulties in UK schools by changing power systems and adapting the approach to non-attendance (Southwell, 2006).

Kearney and Silverman (1990) found that there are four main reasons for school avoidance: (a) to avoid uncomfortable feelings brought on by attending school, such as feelings of anxiety or low mood; (b) to avoid situations that might be stressful, such as academic demands; (c) to reduce separation anxiety or to gain attention from significant others, such as parents or other family members; and (d) to pursue tangible reinforcers outside of school, such as going shopping or playing computer games during school time.

Thus, EBSA seems to be underpinned by complex and interconnected individual, family, and school factors, including perceived ability to cope with academic demands, experiences of bullying, changes in family dynamics, and transitions between school phases (King & Bernstein, 2001; Thambirajah et al., 2008). According to Wimmer (2010), school avoidance is often associated with other difficulties such as separation anxiety, social anxiety, performance anxiety, academic difficulties, and physical symptoms (such as headaches) – factors that can contribute to both the onset *and* the maintenance of school avoidance. Certain events have also been posited to trigger the onset of school avoidance, including death of a loved one, illness of the young person, moving to a new school, as well as transitions such as moving into a new class (Wimmer, 2010).

A notable onset factor identified in the literature is difficulty with peer relationships. Incidents of bullying are common among students who are unable to attend school for a prolonged period (Egger et al., 2003), and it has been suggested that these young people are more likely to present as shy and withdrawn in comparison to their peers (Wimmer, 2010). While victimization from bullying is a primary correlate of school avoidance (Astor et al., 2002), conversely, connectedness with peers and adults can be a protective factor against bullying and, therefore, promotes attendance (Sobba, 2019).

## Supportive Strategies

As discussed, the first stage of intervention involves changing the language used to describe school non-attendance as avoidance underpinned by emotional needs and anxiety about the school system as opposed to defiant behaviour. Such understanding can then inform interventions put in place to help the student return to school (Camden EPS, 2021; Thambirajah et al., 2008; West Sussex EPS, 2018).

When considering support for young people experiencing EBSA, Wimmer (2010) emphasized the importance of early identification and intervention to prevent avoidant behaviour from becoming entrenched. A gradual plan to slowly reintroduce the student back into school, moving through increasingly anxiety-provoking steps (e.g., visiting school when it is closed, going into the reception area of school) is recommended (West Sussex EPS, 2018; Wimmer, 2010). Strategies related to managing anxiety about attending school have also been recommended, including teaching coping strategies, reducing negative self-talk, teaching about the causes of anxiety, and offering safe spaces where students can go to if they are feeling overwhelmed during the school day (Wimmer, 2010). Another factor that has been identified as important for students returning to school is predictability in the classroom in terms of order and organisation so the young person feels secure in what will be happening during the school day (Havik et al., 2015).

Further, the concept of social capital has been suggested as a potential protective factor in promoting school attendance. Social capital is defined as the social networks that groups create to ensure cohesion (Coleman, 1988). Participation in school activities and relationships with peers and adults have been found to counteract the negative effects of bullying and, therefore, inhibit school avoidance (Sobba, 2019). Strategies related to maintaining positive, safe relationships with trusted adults and peers include having a trusted staff member greet the student at the start of the day, providing the student with special roles and responsibilities in school, and helping the student form relationships with a supportive peer who can act as a buddy (Wimmer, 2010).

Teacher-student relationships are an important factor in promoting school attendance in so much as they relate to the students’ perception of the teacher’s ability to manage behaviour in classes (thus reducing bullying). In a study of the features of a specialist setting that encouraged school attendance, Wilkins (2008) found that student-teacher relationships were key in supporting re-engagement with school. Students attending Specialist schools felt that staff were fair, good at listening to why students behaved in a particular way, and focused on understanding why behaviours had occurred rather than responding in a punitive manner. Students, therefore, felt that their teachers cared for them, were flexible in their approach to them, and were available to provide individualised attention when needed. Adapting learning to meet the individual needs of pupils has also been cited as a supportive factor for students attending specialist settings (Raywid, 1994). In contrast, lack of teacher support and fear of the teacher are associated risk factors for school avoidance (Havik et al., 2013, 2015).

The importance of staff working cooperatively with parents to support young people’s education in school is widely accepted. However, the impact of parents on school avoidance is not universally agreed upon. That is, while Wimmer (2010) argued that it is important to work alongside parents to support reintegration back into school, Havik et al. (2015) found that parental interest in school work is only weakly associated with attendance for school avoiders, suggesting that it may be difficult for parents alone to support their children to attend school.

## The Present Study

Although the term EBSA is beginning to be used among some schools and local authority services, it is part of a relatively new understanding of persistent school non-attendance and, therefore, only a small amount of research has studied attendance in terms of emotions and anxiety. And as such, research is also limited in terms of how students experiencing EBSA can be supported to engage in education and attend an educational setting, particularly in UK schools.

The present study was undertaken at a specialist GCSE setting for students who had previously experienced EBSA while attending mainstream school. All students had mental health difficulties as identified by a medical professional, a prerequisite for attending. The study aimed to explore the factors that students valued within this setting, which, for the purpose of anonymity, will subsequently be referred to as School X. The work was commissioned by the programme as part of the author’s role as an educational psychologist, and the outcome was subsequently written up into a study for this paper.

School X uses a particular model, agreed upon by pupils, parents, and the head teacher, that puts an emphasis on young people taking responsibility, with support from adults, for their education. That is, the setting is not simply a haven but a supportive, safe place where the students are expected to put in high levels of effort to develop and grow. Specifically, the aim is for students to develop independence and coping skills and achieve academic qualifications that will benefit them in future.

For three consecutive years, all students in School X completing their GCSEs showed an improvement in attendance and 85% achieved above their predicted grade upon entry to school. In addition, 95% of students were still in post-16 study (for example, attending college or university) after leaving the school. The present study aimed to explore protective factors in a specialist setting where students have previously made progress in terms of attendance and achievement. Specifically, “What are the elements that students value in School X?”

## Method

### Participants

Participants were recruited through opportunity sampling and on a voluntary basis. All students attending the school at the time of the study were asked for their consent to participate. Of the 19 students asked, 12 students took part in the structured, individual interviews and 7 took part in the semi-structured group interview. Some of them took part in both parts of the feedback. Participants were 53% male and 47% female. All were in Year 11 (aged 15 or 16), studying the GCSE curriculum.

### Design

The study used an exploratory single-case study design (Yin, 2003). The intention was to conduct a strengths-based evaluation of the specialist setting for the purpose of understanding which elements were valued by students. All participants received the same questions using the same process.

### Procedure

Two elements were used to inform the questions asked in the quantitative part of the study. The first was information available from previous research relating to EBSA. For the second element, the researcher asked school staff to write an individual, anonymous list of factors they believed were positively impacting GCSE results and attendance rates. As a result of those two sources of information, questions were related to two main themes: relational and academic supports. Both the quantitative and qualitative elements were informed by these pre-identified factors. The questions in the qualitative part of the study were deliberately open-ended to elicit information that might not have been captured by the quantitative information.

In the qualitative part of the study, the seven participants sat in their classroom at enough distance from each other so that they could not confer or see what others were writing. Participants were asked to answer some questions using an anonymised software system. This was done to gain the students’ views in an unprompted way to reduce group influence on their responses. Participants were first told to “Use three words to describe the school.” Next, they were asked, “What is it about this school that you think is positive?”

In the quantitative part of the study, participants met with the researcher individually in a familiar room, separate from the main classroom, to preserve confidentiality. Participants then took part in a Q-sort questioning task in a group situation. The Q-sort technique (McKeown & Thomas, 2013) is a self-contained “qualiquantilogical” methodology used to explore human subjectivity. This technique was used to gain the unique perspectives of each participant in a way that could then be brought together through a ranking system. It prevented the participants from having to articulate their views and provided structure to map their thoughts onto. The stages of the Q methodological study included the following:

1. The researcher generated a set of 38 items (the Q set). All items, or statements, were arranged within two themes: relational elements or academic elements, presented as statements. The statements could be rated by participants in terms of the extent to which they agreed with each. The statements were generated from the overall field of knowledge surrounding the topic and from school practitioners’ comments about what they believed to be effective.
2. The statements were placed on cards for participants to sort on to a Q grid according to specified criteria (e.g., from “most agree/most helpful” to “most disagree/most unhelpful”). Through sorting the cards, participants provided a model of their viewpoint.
3. Each column of the grid represents a discrete numerical rating ranging from zero to four. By sorting the statements, participants assigned a numerical value to each item. The gestalt array of statements produced by participants was analysed to reveal patterns and commonalities in participants’ responses.
4. The top-five ranked statements were identified for each of the themes: relational and academic.

The next part of the analysis involved a combined analysis of both data sets. This included the collated information in the qualitative part of the study and the information gathered through the Q-sort technique in the quantitative part. To analyse the data set, thematic analysis (Braun & Clarke, 2013) was used. An inductive approach was used, although the authors accept that prior knowledge of the area might have influenced the analysis being purely inductive. The process involved the phases of analysis mapped out by Braun and Clarke (2013):

1. Phase one: Initial data were read and considered repeatedly. The data were in written form through the Q-sort results and the information gathered through the software programme from the semi-structured questions.
2. Phase two: Codes were identified, reduced by finding patterns across them, and then compiled.
3. Phase three: Initial themes were identified.
4. Phase four: The extrapolated themes were reviewed to ensure that they were reflective of the data set and made sense generally.
5. Phase five: A thematic map was produced, using overarching and subordinate themes. The themes were then examined to ensure that they were a true reflection of the original data.
6. Phase six: Reporting on the themes and clearly describing how they link with the research question is a key part of thematic analysis. In this paper, the themes are described in the results section.

### Analysis

The quantitative data from the Q-sort activity were analysed; each statement was given a score out of a maximum of 100, and participants’ scores were totaled for each statement. The statements could then be ranked to reflect the most useful elements for most students. Out of the 38 original statements, some statements were not ranked because they were not seen as relevant or were not applicable, which left 24 statements that were ranked across the participant group. The top-five ranked statements were identified for both the pastoral and academic results.

Qualitative data produced from both semi-structured questions were collated through a computer software programme, *Mentimeter* (*www.Mentimeter.com*), that presented the information in visual, clear way.

As already described, data produced from both qualitative and quantitative parts of the evaluation were analysed. It was felt that thematic analysis provided the flexibility and opportunity to elicit rich and detailed patterns across the data produced from the Q-sort evaluation and from the semi-structured questions.

## Results

### Quantitative Results

**Figure 1:**
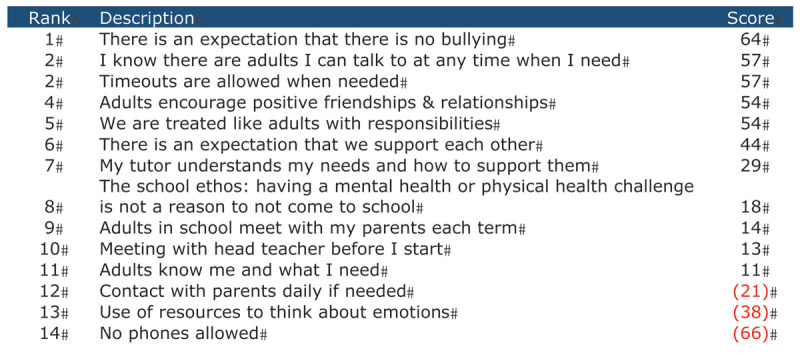
Participants’ Rankings of Relational Elements.

**Figure 2:**
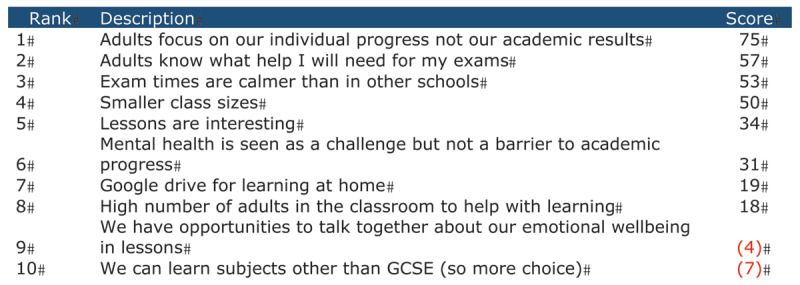
Participants’ Rankings of Academic Elements.

The quantitative results are outlined first. Results are presented in order of how highly they were ranked and remain separated into relational and academic elements as they were presented to participants originally. ***Figures 1*** and ***2*** present the statements ranked by the individual participants and how they were collectively ranked. The higher the number, the more value the participants placed on the statements as a collective. The top five of all the statements ranked are represented within the findings because these were found to be most important to the participants.

#### Relational Elements

***Figure 1*** shows how statements were ranked for the relational elements. The top-five ranked viewed as most helpful by the participants were:

- There is an expectation that there is no bullying
- Timeouts are allowed when needed
- I know there are adults I can talk to when I need
- Adults encourage positive friendships and relationships
- We are treated like adults with responsibilities

#### Academic Elements

***Figure 2*** lists the rankings of the statements related to the academic elements. The top-five viewed as most helpful by the participants were:

- Adults focus on individual progress not our academic results
- Exam times are calmer than in other schools
- Adults know what will help me in my exams
- Smaller class sizes
- Lessons are interesting

### Qualitative Results

The participants were first requested to “Use three words to describe School X.” The software used automatically produces a word cloud and increases the size of words the more times they are written; for example, the most frequently stated word was “supportive,” followed by “helpful” and “kind” as the second and third most stated words, respectively. Participants were also asked: “What is it about this school that you think is positive?” Again, their answers were collated automatically in a clear and visual way by the computer software programme.

The qualitative data and the five most helpful statements from the pastoral and academic Q-sort results were analysed by way of thematic analysis (Braun & Clarke, 2013) to produce two overarching themes: interconnectivity and psychological safety.

### Summary of Results

**Figure 3:**
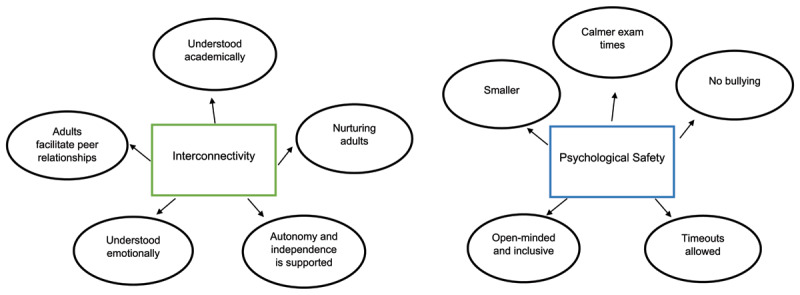
Thematic analysis of qualitative and quantitative data: overarching and subordinate themes.

***Figure 3*** maps out all the results into overarching and subordinate themes using results from all the data gathered. The overarching theme of interconnectivity shows the value placed on the relational aspects of the setting. Participants valued adults who displayed nurturing characteristics, who facilitated peer relationships, and who supported them to become autonomous and independent. A clear value was placed on being understood and valued personally, emotionally, and academically. Participants recognised when adults in the setting understood their learning style and could make lessons interesting for them.

The second overarching theme shows the significance of safety because of the importance placed on an anti-bullying ethos. The importance of safety is also evident through the participants placing high value on being able to access safe spaces for a timeout. Smaller class sizes, a calm exam environment and engaging lessons also suggest that participants valued the sense of safety created by the school to enable them to learn.

## Discussion

The present study explored the factors that students value in a specialist setting where students have previously made progress in terms of attendance and attainment. Interviews with participants and analysis of questionnaire data demonstrated that the strategies employed within the setting that were most valued by students fell into two overarching themes: interconnectivity and psychological safety.

### Interconnectivity

Within the theme of interconnectivity and relationships within School X, participants pointed to factors such as nurturing adults being available to talk to about their concerns, adult support to promote peer relationships, and positive, trusted relationships with adults, which have also been identified in previous research (Wimmer, 2010). Participants seemed to value that school staff understood them both academically and emotionally and, therefore, were able to form these relationships and respond to them effectively.

As suggested by previous research, difficulties in peer relationships and bullying are common among students who struggle to attend school (Astor et al., 2002; Egger, 2003). Promoting social capital by helping students to form relationships with peers and counteracting bullying has been found to be important for promoting attendance (Sobba, 2019; Wimmer, 2010), and is also evident in the present study, suggesting that having a sense of social connectedness, and therefore safety, was important for participants.

One of the ways in which staff at School X may have formed positive relationships was through the responsibility given to students. Thus, participants valued that adults gave them a sense of responsibility within school and recognised their maturity, giving them a feeling of control and autonomy, something they may have not felt due to anxiety about attending school previously (see Thambirajah et al., 2008). The fact that all participants were approaching the end of compulsory schooling, aged 15 or 16, may also explain why this more adult treatment was valued highly.

Our findings are similar to those of Wilkins (2008) regarding specialist settings, demonstrating that adult-student relationships were key factors to promote school attendance. Wilkins (2008) found that participants benefitted from receiving individualised attention from adults who they felt cared for them and were flexible in how they supported them. These elements seemed to also be valued by participants in the present study.

Learning and academic progress happens within the context of positive relationships suggesting that relational experiences are important for learning. As Greenhalgh (1994) argues, capacity for learning and relationship building are closely related, suggesting that relationships are crucial for academic progress. The availability of trusted adults who were consistent attachment figures within the school environment may have enabled the students to be more available for learning. School X’s approach also provides students with responsibility to sensitively challenge them to develop strategies to manage anxiety (Kearney & Bensaheb, 2006), which may have been possible due to the relationships formed between staff and students. Thus, the theme of interconnectivity supports the idea that relationships are valued by students and underpin positive academic experiences.

### Psychological Safety

Participants also valued the sense of psychological safety created by School X. Specifically, they felt that this was achieved through having available safe spaces for students to use if they were feeling overwhelmed, the expectation that there is no bullying at school, the use of timeouts to support students to regulate themselves when they feel overwhelmed, smaller class sizes, carefully planned, engaging lessons, and an approach to exams that takes individuals needs into consideration. The presence of adults who were open-minded and inclusive, for example, by not stigmatising mental health, was also cited as a helpful factor, perhaps suggesting that participants were enabled to feel secure at school because they were accepted and understood by the adults around them.

Previous research has found that avoidance of stressful situations such as academic demands and perceived inability to cope are factors that can push students away from attending school (Kearney & Silberman, 1990; Thambirajah et al., 2008). Anxiety around academic performance has also been cited as a contributing factor for EBSA (Wimmer, 2010). In the present study, participants cited support with exams as important factors in supporting them to attend school. Specifically, they seemed to value the carefully planned, personalised, and calm approach to examinations perhaps because it supported them to manage anxiety in situations that they have previously found stressful. In support of previous research into specialist settings (Raywid, 1994), the present study also found that small class sizes and adapting lessons to ensure that they are engaging and interesting was a valued part of students’ educational experience, perhaps because these factors made them feel more secure and capable in lessons.

Interpersonal, relational benefits and supports to create a sense of psychological safety are valued by students, and these factors are connected and dependent upon each other. Relationships have been highlighted as crucial factors in creating capacity for learning, the suggestion being that students with a secure base in the form of a supportive, trusted adult at school feel safe and contained and can, therefore, engage in the challenge of learning and assessments. It has been argued that, in order to promote learning, schools must focus first on creating safe relationships within school (Greenhalgh, 1994; Youell, 2006), something that students at School X appear to value highly. It is likely that the calm approach to examinations or personalised learning available to students at School X would not be possible without strong, relational foundations and that these relationships would not be formed if academic supports were not as individualised or readily available. Thus, the model used by School X seemed to communicate to students that they were important to the adults supporting them, who gave them responsibility, recognised what they needed to feel calm, listened to them, and adapted instruction to meet their needs. The current study argues that, although learning supports should be put in place for students experiencing EBSA, without sufficient, supportive adult-student and student-student relationships, these structures are unlikely to create full engagement within school as the latter were so highly valued by participants.

### Implications for Practice

The current study has implications for strategies and approaches employed within specialist settings for students who present with EBSA. Our findings suggest that it is the school structures and systems that can bring about change in terms of school attendance as opposed to factors within the students themselves (Southwell, 2006). In line with Thambirajah et al.’s (2008) suggestion that intervention can be planned based on the factors that push young people away from school and pull them towards home, the present study identified a range of protective factors that can overcome some of these difficulties and promote school attendance.

Although conducted within a specialist setting, the study also has implications for approaches employed within mainstream schools. Although some strategies may not be possible in all settings (such as smaller class sizes), the access to trusted adults, use of safe spaces, emphasis on creating supportive peer relationships, and a calm approach to examinations could be considered as part of reintegration plans for students who have experienced EBSA in mainstream schools. The underlying principles of a programme that creates a sense of psychological safety and interconnectivity could underpin approaches used by all settings when students present with EBSA.

### Limits of the Study

The current study was implemented by an educational psychologist (EP) linked to the setting at the request of the head teacher, who had noticed positive associations with the model being used (e.g., on GCSE results and attendance). Consequently, there are several significant limitations. Of note is the likelihood of researcher bias, in that the EP was aware of the positive associations related to the approach used in the school and might unwittingly have created bias within the data gathering through, for example, tone of voice or body language. Unconscious researcher bias could also have occurred within the analysis phase when the researcher might have been drawn to data that proved the hypothesis.

There is also a high probability of issues relating to internal validity. The EP had working relationships with the adults in the setting and had also worked previously with some of the students. This may have created a bias, particularly for the adult participants, as they may have wanted to speak positively about the programme to please the EP or the head teacher. The Q-sorting technique was delivered in a strict format individually for each participant, and care was taken to ensure the experience was the same for each participant; however, slight differences might have occurred that could have had an impact on reliability between participants. The two group elements of the research could be subject to biases, in particular group bias, in which the participants might have been swayed by what others were writing.

Finally, it is important to acknowledge that the findings of this study are based on a small sample size and as such are potentially transferable but not generalizable; they provide an insight into practices that could be explored further in future research. The analysis was also based on brief and succinct qualitative data and, although thematic and content analysis were used, more in-depth information could have been gathered from individual interviews with students.

### Implications for Further Research

The study explored factors valued by students attending a specialist setting for students who have struggled to engage with school due to mental health difficulties. Future research could incorporate the views of parents and school staff to provide a richer picture of the factors that promote school attendance. It might also be helpful to explore the approaches of other alternative programmes in order to compare strategies employed and look for consistency across them.

In addition, participants in the current study were all in Year 11 (aged 15 or 16), which may have accounted for some of the findings discussed here. Students seemed to value the guidance and support offered by trusted adults in a safe environment where they felt respected and treated like adults. Younger children, who are less autonomous learners and at a different educational and psychological stage of development, may make different suggestions about how school could support them, and it would therefore be helpful to explore their views.

Future research could also consider how students who have experienced EBSA can be encouraged to integrate into a mainstream setting as, due to the difference in structure and resources, it is likely that some of the approaches may be operationalised differently within this context. Finally, the present study did not explore the correlation between approaches used at School X and academic progress as these data were not gathered as part of the study to respond to the research question. Future research could explore academic progress for students experiencing EBSA who attend specialist settings of a similar nature.

## Conclusion

The present study explored the protective factors valued by participants attending a specialist setting where students had previously made progress in terms of attendance and academic outcomes. The importance of interconnectivity and psychological safety were found to be most important to students, factors that, it is argued, are linked and dependent upon each other. Through the factors identified, School X’s model seemed to communicate to students that they were valued, understood, and important to the staff working with them; Characteristics that then create the safe base needed by students who have previously struggled with school attendance to feel confident to engage in school and learning.

Previous studies have found that the outcomes for students experiencing EBSA are generally poor, including negative impact on academic attainment, employment, mental health difficulties, and social engagement (Garry, 1996; Pellegrini, 2007; Taylor, 2012; Walter et al., 2010). Findings from the current study present a model for supporting students who avoid school to enable them to not only attend an educational programme but engage in learning and academic qualifications. Participants reported valuing supportive adults who understood them as individuals and responded to their needs. Prioritising interconnectivity and psychological safety in any educational programme could, therefore, contribute to the prevention of further negative life outcomes for students experiencing EBSA.

## References

[1] Astor, R. A., Benbenishty, R., Zeira, A., & Vinokur, A. (2002). School climate, observed risky behaviors, and victimization as predictors of high school students’ fear and judgments of school violence as a problem. Health Education & Behavior, 29(6), 716–736. DOI: 10.1177/109019802237940 12456131

[2] Bjarnason, T., & Thorlindsson, T. (1994). Manifest predictors of past suicide attempts in a population of Icelandic adolescents. Suicide and Life-Threatening Behavior, 24(4), 350–358. 7740593

[3] Braun, V., & Clarke, V. (2013). Successful qualitative research: A practical guide for beginners. Sage.

[4] Coleman, J. S. (1988). Social capital in the creation of human capital. American Journal of Sociology, 94, 95–120. DOI: 10.1086/228943

[5] Egger, H. L., Costello, J., & Angold, A. (2003). School refusal and psychiatric disorders: A community study. Journal of the American Academy of Child & Adolescent Psychiatry, 42(7), 797–807. DOI: 10.1097/01.CHI.0000046865.56865.79 12819439

[6] Elliott, J. G. (1999). Practitioner review: School refusal: Issues of conceptualisation, assessment, and treatment. Journal of Child Psychology & Psychiatry, 40(7), 1001–1012. DOI: 10.1111/1469-7610.00519 10576531

[7] Finning, K., Ukoumunne, O. C., Ford, T., Danielson-Waters, E., Shaw, L., Romero De Jager, I., & Moore, D. A. (2019). The association between anxiety and poor attendance at school–a systematic review. Child and Adolescent Mental Health, 24(3), 205–216. DOI: 10.1111/camh.12322 32677217

[8] Frydman, J. S., & Mayor, C. (2017). Trauma and early adolescent development: Case examples from a trauma-informed public health middle school program. Children & Schools, 39(4), 238–247. DOI: 10.1093/cs/cdx017

[9] Garry, E. (1996). Truancy: First steps to a lifetime of problems. Washington DC Office of Juvenile Justice and Delinquency Protection. DOI: 10.1037/e306412003-001

[10] Greenhalgh, P. (1994). Emotional growth and learning (1st ed.). Routledge. DOI: 10.4324/9780203424681

[11] Gulliford, A., & Miller, A. (2015). Coping with life by coping with school? School refusal in young people. In T. Cline, A. Gulliford, & S. Birch (Eds.), Educational psychology (pp. 283–305). Routledge. DOI: 10.4324/9781315719962

[12] Havik, T., Bru, E., & Ertesvåg, S. K. (2015). School factors associated with school refusal-and truancy-related reasons for school non-attendance. Social Psychology of Education, 18(2), 221–240. DOI: 10.1007/s11218-015-9293-y

[13] Heyne, D., & Rollings, S. (2002). School refusal. Blackwell.

[14] Kearney, C. A., & Benseheb, A. (2006). School absenteeism and school refusal behavior: A review and suggestions for school-based health professionals. Journal of School Health, 76(1), 3–7. DOI: 10.1111/j.1746-1561.2006.00060.x 16457678

[15] Kearney, C. A., & Silvermann, W. K. (1990). A preliminary analysis of afunctional model of assessment and treatment of school refusal behaviour. Behaviour Modification, 14, 340–366. DOI: 10.1177/01454455900143007 2375736

[16] King, N., & Bernstein, G. (2001). School refusal in children and adolescents: A review of the past 10 years. Journal of American Academy of Child Adolescent Psychiatry, 40(2), 197–205. DOI: 10.1097/00004583-200102000-00014 11211368

[17] London Borough of Camden. (2021). Emotionally based school avoidance. Available at download.asp (royalfree.camden.sch.uk)

[18] McKeown, B., & Thomas, D. B. (2013). Q methodology (Vol. 66). Sage Publications. DOI: 10.4135/9781483384412

[19] Pellegrini, D. (2007). School non-attendance: Definitions, meanings, responses, interventions. Educational Psychology in Practice, 23(1), 63–77. DOI: 10.4135/9781483384412

[20] Planty, M., DeVoe, J. F., Owings, J. A., & Chandler, K. (2005). An examination of the conditions of school facilities attended by 10th-grade students in 2002 (ED TAB. NCES 2006–302). National Center for Education Statistics.

[21] Raywid, M. A. (1994). A school that really works: Urban academy. The Journal of Negro Education, 63(1), 93–110. DOI: 10.2307/2967333

[22] Sobba, K. N. (2019). Correlates and buffers of school avoidance: A review of school avoidance literature and applying social capital as a potential safeguard. International Journal of Adolescence and Youth, 24(3), 380–394. DOI: 10.1080/02673843.2018.1524772

[23] Southwell, N. (2006). Truants on truancy – A badness or a valuable indicator of unmet special educational needs? British Journal of Special Education, 33(2), 91–97. DOI: 10.1111/j.1467-8578.2006.00420.x

[24] Taylor, C. (2012). Improving attendance at school. London DFE.

[25] Tobias, A. (2019). A grounded theory study of family coach intervention with persistent school non-attenders. Educational Psychology in Practice, 35(1), 17–33. DOI: 10.1080/02667363.2018.1518215

[26] Thambirajah, M. S., Grandison, K. J., & De-Hayes, L. (2008). Understanding school refusal: A handbook for professionals in education, health and social care. Jessica Kingsley Publishers. DOI: 10.7748/mhp.12.3.22.s22

[27] Walter, D., Hautmann, C., Rizk, S., Patermann, M., Sinzig, J., Lehnmuhl, G., & Doepfner, M. (2010). Short term effects of impaired cognitive behavioural treatments of adolescents with anxious-depressed school absenteeism: An observational study. European Child and Adolescent Psychiatry, 19, 835–844. DOI: 10.1007/s00787-010-0133-5 20835738

[28] West Sussex Educational Psychology Service. (2018). Emotionally based school avoidance. | www.schools.westsussex.gov.uk

[29] Wilkins, J. (2008). School characteristics that influence student attendance: Experiences of students in a school avoidance program. The High School Journal, 12–24. DOI: 10.1353/hsj.2008.0005

[30] Wimmer, M. (2010). School refusal: Information for educators. Helping children at home and school III: Handouts for educators. Bethesda, MD: National Association of School Psychologists.

[31] Yin, R. K. (2003). Case study research: Design and methods (3rd ed.). Sage. DOI: 10.3138/cjpe.30.1.108

[32] Youell, B. (2006). The learning relationship: Psychoanalytic thinking in education. Routledge. DOI: 10.4324/9780429482281

